# Frequencies and reasons for unplanned emergency department return visits by older adults: a cohort study

**DOI:** 10.1186/s12877-023-04021-x

**Published:** 2023-05-18

**Authors:** Merel van Loon-van Gaalen, Ilje E. Voshol, M. Christien van der Linden, Jacobijn Gussekloo, Roos C. van der Mast

**Affiliations:** 1 Emergency Department, Haaglanden Medical Center, P.O. Box 432, 2501 CK The Hague, The Netherlands; 2 Department of Psychiatry, Leiden University Medical Center, Leiden, The Netherlands; 3 GeriCall, The Hague, The Netherlands; 4 Department of Internal Medicine, Section of Gerontology and Geriatrics, Leiden University Medical Center, Leiden, The Netherlands; 5 Department of Public Health and Primary Care, Leiden University Medical Center, Leiden, The Netherlands; 6 Department of Psychiatry, CAPRI-University Antwerp, Antwerp, Belgium

**Keywords:** Older patients, Emergency department, Geriatric, Unplanned return, Return presentations

## Abstract

**Background:**

As unplanned Emergency Department (ED) return visits (URVs) are associated with adverse health outcomes in older adults, many EDs have initiated post-discharge interventions to reduce URVs. Unfortunately, most interventions fail to reduce URVs, including telephone follow-up after ED discharge, investigated in a recent trial. To understand why these interventions were not effective, we analyzed patient and ED visit characteristics and reasons for URVs within 30 days for patients aged ≥ 70 years.

**Methods:**

Data was used from a randomized controlled trial, investigating whether telephone follow-up after ED discharge reduced URVs compared to a satisfaction survey call. Only observational data from control group patients were used. Patient and index ED visit characteristics were compared between patients with and without URVs. Two independent researchers determined the reasons for URVs and categorized them into: patient-related, illness-related, new complaints and other reasons. Associations were examined between the number of URVs per patient and the categories of reasons for URVs.

**Results:**

Of the 1659 patients, 222 (13.4%) had at least one URV within 30 days. Male sex, ED visit in the 30 days before the index ED visit, triage category “urgent”, longer length of ED stay, urinary tract problems, and dyspnea were associated with URVs. Of the 222 patients with an URV, 31 (14%) returned for patient-related reasons, 95 (43%) for illness-related reasons, 76 (34%) for a new complaint and 20 (9%) for other reasons. URVs of patients who returned ≥ 3 times were mostly illness-related (72%).

**Conclusion:**

As the majority of patients had an URV for illness-related reasons or new complaints, these data fuel the discussion as to whether URVs can or should be prevented.

**Trial registration:**

For this cohort study, we used data from a randomized controlled trial (RCT). This trial was pre-registered in the Netherlands Trial Register with number NTR6815 on the 7^th^ of November 2017.

**Supplementary Information:**

The online version contains supplementary material available at 10.1186/s12877-023-04021-x.

## Introduction

With demographic change, there is an increase in Emergency Department (ED) presentations by patients aged 70 years and older worldwide [1]. Up to 25% of these older patients have an unplanned ED return visit (URV) within one month [2–6]. Since URVs in older adults are associated with adverse health outcomes, they are often viewed as negative [3, 7]. Therefore, many EDs have initiated post-discharge interventions in order to reduce URVs. [8, 9]

Many post-ED discharge intervention programs are focused on older patients at high risk for hospital return. However, prediction tools that have been developed to identify patients at risk have poor predictive accuracy, contain different predictors, and are often not suitable for clinical use [4, 10–13]. However, all previous studies consistently report that the majority of older adults who return to the ED suffer from chronic and often comorbid health conditions, functional dependency or cognitive problems [2, 10, 14, 15]. In addition, several (psycho)social factors, such as living alone, lack of social support and uncertainty about the health condition, as well as insufficient understanding or provision of discharge information are found to be associated with URVs in older adults. [2, 6, 7, 11, 14–19]

Several of these predicting factors could be addressed through specific interventions, such as patient education and community follow-up by a geriatric nurse. However, systematic reviews evaluating the effects of post-discharge interventions initiated in the ED have found that many were not effective in reducing ED re-attendances [8, 9]. In a pragmatic randomized controlled trial, our research group also failed to find a beneficial effect of a transitional care program, consisting of post-ED discharge telephone follow-up for older adults, on the reduction of unplanned hospital admissions and URVs within 30 days after ED discharge [20].

In order to understand why these interventions are not effective in reducing URVs, more insight is needed into the reasons why older patients return to the ED. Therefore, we investigated the frequencies, associated patient and ED visit characteristics and reasons for URVs within 30 days after the index ED visit among patients aged ≥ 70 years. In addition, we examined whether specific categories of reasons for URVs were associated with the number of URVs per patient.

## Methods

### Study design and setting

For this study, we used data from a pragmatic randomized controlled trial (RCT). The research question of this RCT was whether a telephone follow-up call reduces unplanned hospitalizations and URVs within 30 days of ED discharge, compared to a satisfaction survey call. The trial was conducted in the EDs of Haaglanden Medical Center (HMC), a non-academic teaching hospital in the Netherlands, from February 1, 2018 to July 1, 2019. In this RCT, 3175 patients were allocated to either the intervention (*n* = 1516) or the control (*n* = 1659) group, according to the month of their ED visit; patients included in odd months received an intervention telephone call to identify post-discharge problems and to offer additional information, and patients included in even months received a satisfaction survey telephone call [20]. The Medical Ethics Review Committee of HMC waived the necessity for formal approval of the study as it closely followed routine care (METC Zuidwest Holland, nr. 17–028).

### Patients

For this study, only observational data from control group patients were used to exclude a possible effect of the intervention telephone follow-up call. Patients aged ≥ 70 years who were discharged from one of the EDs of HMC to an unassisted living environment were eligible for inclusion. Exclusion criteria were: admission to the hospital, discharge to a nursing home, another care facility or assisted living environment, and planned follow-up appointment at an outpatient clinic or at the ED within 24 h [20].

### Data collection and measurements

#### Unplanned ED return visits (URVs)

Data on ED return visits were collected from the electronic hospital system (EHS). ED return visits that could not be foreseen were defined as URVs [21]. The index ED visit was the first ED visit during the study period that was followed by a telephone call.

#### Baseline data

We used baseline data that were associated with URVs in previous studies, including demographics (age [4, 10, 13], gender [4, 5, 10], whether or not living alone [2, 3, 11, 22],) and ED visit characteristics (mode of arrival, Manchester Triage System triage urgency level [23], chief complaint, ED length of stay [2, 10, 11, 24]). We also used data concerning level of ED crowding at discharge, measured by the National Emergency Department OverCrowding Scale (NEDOCS) [25]. Data were abstracted from the EHS by an information technology specialist, who was not involved in the study [20].

#### Determination of reasons for URVs

Prior to the start of the study, reasons for URVs were defined and categorized, based on findings in the literature (see Additional file 1) [4, 7, 15, 19, 26]. Two investigators (MvLvG and IEV), both medical doctors, independently determined and categorized the reason for each URV by reviewing the emergency medical records (EMRs). In case of disagreement, the EMR was reviewed and reasons for ED return were discussed until consensus was achieved. In case of no agreement, the EMR was reviewed by a third investigator (MCvdL) for the final decision. This study method has been used in previous studies on URVs [16, 26–28]. During analyses, we found that only few URVs were categorized as physician-related, system-related or not classifiable. Therefore, these three categories have been merged into the “other reasons” category. This resulted in the following four main categories: 1. patient-related reasons, 2. illness-related reasons, 3. new complaints and 4. other reasons (see Additional file 1). The study was conducted in adherence to the Strengthening the Reporting of Observational Studies in Epidemiology (STROBE) Statement [29].

### Statistical analysis

Categorical data are presented as numbers and percentages. Continuous data were skewed and therefore presented as median and interquartile ranges (IQR). Differences in characteristics of patients with and without URVs were analyzed using X^2^-tests and univariable logistic regression.

The X^2^-test was used to examine the association between number of URVs per patient and the categories of reasons for URVs. Odds ratios (ORs) were calculated with 95% confidence intervals (95% CIs). If a patient had multiple URVs during the 30-day follow up period, only the first URV was included to determine the reason for unplanned return and to assess associations between patient and index ED visit characteristics and occurrence of an URV. To investigate whether specific categories of reasons for URVs were associated with the number of URVs per patient, all URVs within 30 days after the index ED visit were included in the analysis.

Inter-rater reliability regarding the initial determination of reasons and categories of URVs was measured with Cohen’s kappa coefficient.

Statistical analyses were performed using the Statistical Package for the Social Sciences (IBM Corp. Released 2019. IBM SPSS Statistics, Version 26.0. Armonk, NY, USA).

## Results

Of the 1659 patients, 222 (13.4%) had at least one URV within 30 days. The total number of URVs within 30 days was 279.

### Patient and ED visit characteristics associated with URVs

Table 1 shows the differences in baseline patient and index ED visit characteristics between patients with and without an URV. In univariate analysis, the following factors were associated with an URV within 30 days: male sex, ED visit in the 30 days before the index ED visit, triage category “urgent”, longer length of ED stay, and the chief complaints “urinary tract problems”, and “dyspnea”.

**Table 1:** Baseline patient and index Emergency Department visit characteristics of patients with and without an URV

	Unplanned ED return visit (URV) ≤ 30 days		
	Yes (*n* = 222)	No (*n* = 1437)	OR (95% CI)
Demographics			
Age in years, median (IQR)	78 (73–83)	78 (73–83)	1.0 (1.0–1.0)^a^
Male sex, n (%)	106 (47.7)	588 (40.9)	1.3 (1.0–1.8)
Living without partner, n (%)^b^	83 (42.3)	413 (37.7)	1.2 (0.9–1.7)
Characteristics of index ED visit			
Mode of referral, n (%)			
- Self-referral	52 (23.4)	307 (21.4)	1.1 (0.8–1.6)
- General practitioner	65 (29.3)	485 (33.8)	0.8 (0.6–1.1)
- Medical specialist	44 (19.8)	246 (17.1)	1.2 (0.8–1.7)
ED visit ≤ 30 days before index visit, n (%)	45 (20.3)	164 (11.4)	2.0 (1.4–2.8)
Arrival by ambulance, n (%)	79 (35.6)	476 (33.1)	1.1 (0.8–1.5)
Triage category urgent, n (%)^c^	168 (76.4)	999 (70.0)	1.4 (1.0–1.9)
ED visit at daytime, n (%)	153 (68.9)	1009 (70.2)	0.9 (0.7–1.3)
Length of ED stay (minutes), median (IQR)	179 (128–242)	151 (106–204)	1.0 (1.0–1.1)^ad^
NEDOCS at discharge ≥ 60, n (%)^e^	66 (34.6)	425 (32.9)	1.1 (0.8–1.5)
Chief complaint, n (%)			
- Urinary tract problems	16 (7.2)	47 (3.3)	2.3 (1.3–4.1)
- Headache or neurological problems	10 (4.5)	55 (3.8)	1.2 (0.6–2.4)
- Wounds	11 (5.0)	76 (5.3)	0.9 (0.5–1.8)
- Abdominal pain	16 (7.2)	76 (5.3)	1.4 (0.8–2.4)
- Syncope or palpitations	8 (3.6)	90 (6.3)	0.6 (0.3–1.2)
- Dyspnea	29 (13.1)	116 (8.1)	1.7 (1.1–2.6)
- Malaise	19 (8.6)	131 (9.1)	0.9 (0.6–1.5)
- Chest pain	28 (12.6)	177 (12.3)	1.0 (0.7–1.6)
- Limb complaints	37 (16.7)	299 (20.8)	0.8 (0.5–1.1)
- Fall or trauma	48 (10.7)	358 (13.1)	0.8 (0.6–1.1)
- Other complaints	17 (7.7)	152 (10.6)	0.7 (0.4–1.2)

*ED* Emergency department, *NEDOCS* National emergency department overcrowding scale, *IQR* Interquartile range, *n* number, *URV* Unplanned emergency department return visit

^a^ In univariable logistic regression model

^b^ Living condition unknown in 26 patients with URV and in 341 patients without URV

^c^ Triage category urgent: red, orange and yellow according to Manchester Triage System. Triage category missing in 2 patients with URV and in 10 patients without URV

^d^ Per 10 min increase in length of stay; OR value of 1.0 is due to rounding

^e^ If the NEDOCS at discharge is ≥ 60, the ED is considered to be busy. NEDOCS at discharge was missing in 31 patients with URV and 144 patients without URV, due to technical malfunction of electronic hospital system on days that patients were discharged from the ED

### Reasons for unplanned ED return

**Fig. 1:**
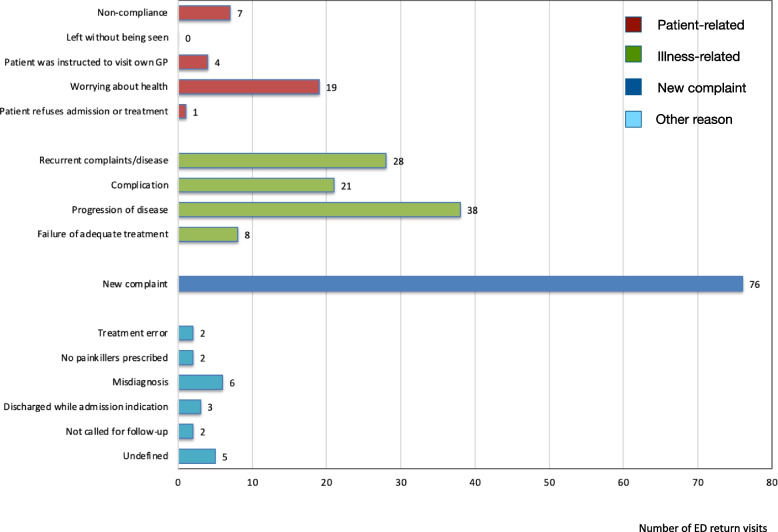
Reasons for unplanned Emergency Department (ED) return visits (*n* = 222), divided into four categories

Figure 1 shows the number of URVs per reason for return. Patient-related reasons for URVs were found in 31 (14%) of the 222 patients with one or more URVs. The two most frequently occurring patient-related reasons for URVs were non-compliance with discharge instructions (*n* = 7), and worrying about health (*n* = 19). Illness-related reasons for URVs were found in 95 (43%) of the 222 patients, of which recurrent complaints/disease (*n* = 28) and progression of disease (*n* = 38) were the two largest subgroups. A new complaint was the reason for URV in 76 (34%) of the 222 patients, and 20 (9%) out of the 222 patients had an URV for other reasons. Within the latter category, 6 of the 20 patients were misdiagnosed during the index ED visit, resulting in inappropriate treatment. Other physician-related and system-related reasons occurred in < 2% of the 222 patients. Five URVs could not be classified and were therefore coded as “undefined”.

### Frequent URVs and reasons for ED return

Of the 222 patients with URVs, 176 (79.2%) had one URV, 39 (17.6%) had two URVs and 7 (3.2%) had three or more URVs within 30 days (Table 2). Most URVs in patients with one or two URVs were illness-related (40.9% and 46.2%, respectively) or because of a new complaint (38.0% and 30.8%, respectively). Patients with three or more URVs also returned mainly for illness-related reasons (72.0%), followed by patient-related reasons (24.0%), while new complaints were less common (4.0%).

**Table 2:** Association between the number of URVs per patient and per category of reasons for URVs

	Number of URVs per patient			
	1	2	≥ 3	Total
Number of patients, n (%)	176 (79.2)	39 (17.6)	7 (3.2)	222
Total number of URVs, n (%)	176 (63.1)	78 (28.0)	25 (9.0)	279
Category reasons for URV:				
Patient-related, n (%)	22 (12.5)	10 (12.8)	6 (24.0)	38
Illness-related, n (%)	72 (40.9)	36 (46.2)	18 (72.0)	126
New complaint, n (%)	67 (38.0)	24 (30.8)	1 (4.0)	92
Other, n (%)	15 (8.5)	8 (10.3)	0 (0.0)	23

*ED* Emergency department, *n* number, *URV* Unplanned emergency department return visit

### Inter-rater reliability regarding assessment of reasons and categories for URVs

The inter-rater reliability after initial independent determination and categorization of the reasons for the URVs, measured with Cohen’s kappa coefficient, was 0.57. All disagreements concerning the determination of the reasons for URVs were solved by discussion between the two researchers and hence, the judgement of a third researcher for the final decision was not needed.

## Discussion

In this study, we found that 222 of the 1659 (13.4%) older adults had at least one URV within 30 days after being discharged from the ED. Of them, 171 (77%) returned for medical reasons, including 95 (43%) for illness-related problems and 76 (34%) for new medical complaints unrelated to the presenting problem of the index ED visit. URVs for patient-related reasons occurred in only 31 (14%) patients. Also, patients with more than one URV returned mainly for illness-related reasons.

The URV rate in our study was comparable with URV rates among older adults reported in other studies [2–4, 10, 30]. We also found that male sex [2, 4, 14, 15], an ED visit in the 30 days before the index ED visit [2, 10, 11, 14], triage category “urgent”, and a longer length of ED stay [24] were more common in patients with an URV. In accordance with other studies, we found that the chief complaints “urinary tract problems” [26], and “dyspnea” [15, 19, 26, 31] were associated with URVs.

Although transitional care programs that focus on patient education and post-discharge support may have a positive effect on the patient’s capacity for self-care, disease control and perceived support [32–34], the limited number of patient-related URVs found in our study may explain why many of these programs do not reduce URVs. Our finding that most older adults returned to the ED for illness-related reasons or new problems suggests that a substantial number of patients needed diagnostic work-up of their health problem and/or acute care. This fuels the discussion of whether URVs can and need to be prevented. If the aim is to divert older patients from the ED, it will have to be sorted out where else diagnostic work-ups can be performed and patients can receive the necessary (acute) care outside the ED. This will depend on the organization of the health care system and should therefore be investigated locally. An example is the organization of an acute geriatric community hospital for older adults [35]. On the other hand, as the ED is organized and equipped to conduct targeted diagnostic work-ups and deliver acute care, it may be more feasible to make existing EDs more senior-friendly by applying the initiatives already described [36–39]. Interventions focusing on close collaboration between primary care, hospital care, and community services may be more successful in reducing unplanned ED visits for older adults than interventions involving only the ED. Within these collaborations, it may be easier to deliver the best care for the patient at the most suitable location. It would be interesting to explore such collaborations in future studies [40, 41].

### Strengths and limitations

We were able to compare an extensive set of patient and ED visit characteristics between patients with and without URVs. Although previous studies mentioned reasons for URVs in older adults, this is one of the few studies that investigated the frequencies of the different reasons for URVs in older adults [2, 7]. Data were prospectively collected and derived from the hospital database to diminish confounding by recall bias.

Some limitations, however, could be considered. The reasons for URVs were defined and categorized prior to the start of the study and based on explicit criteria, used in previous studies. However, the reasons for URVs were determined retrospectively. By having the URVs assessed by two independent researchers, we tried to comply with the classification criteria as much as possible. The Cohen’s kappa coefficient of 0.57, reflecting a moderate inter-rater reliability of the categorization system, may be a limitation.

Furthermore, not all data about health determinants that are associated with hospital return were available. Finally, this study was conducted in two EDs of a non-academic hospital in the Netherlands. The findings may not be generalizable to all EDs. However, two studies, one conducted in a Dutch academic ED and one in two Australian large referral hospital EDs, reported comparable percentages of URVs for illness-related and patient-related reasons [7] and for new complaints [2].

## Conclusion

In this study, most older patients returned unplanned to the ED for medical reasons, whereas URVs for patient-related reasons, such as uncertainty about health or misunderstanding of discharge instructions, were less common. These findings may explain why many transitional care programs that focus on patient education and post-discharge support are ineffective in reducing URVs. In addition, the results suggest that most patients who return to the ED require urgent care. This fuels the discussion as to whether URVs can or need to be prevented.

## Supplementary Information

**Additional file 1.** Definitions of reasons used to analyze unplanned emergency department return visits (URV) and categorization of the reasons.

## Data Availability

The dataset used and analyzed during the current study is available from the corresponding author on reasonable request and with permission of the Board of Directors of Haaglanden Medical Center.

## Abbreviations

ED: Emergency department
URV: Unplanned emergency department return visit
RCT: Randomized controlled trial
HMC: Haaglanden medical center
n: Number
METC: Medisch ethische toetsings commissie
EHS: Electronic hospital system
NEDOCS: National emergency department overcrowding scale
EMR: Emergency medical record
STROBE: Strengthening the reporting of observational studies in epidemiology
IQR: Interquartile ranges
OR: Odds ratio
CI: Confidence interval
