# Supplementary material for: Frequencies and reasons for unplanned emergency department return visits by older adults: a cohort study
Source: BMC Geriatr. 2023 May 18;23:309. doi: 10.1186/s12877-023-04021-x (PMC10193595; doi:10.1186/s12877-023-04021-x)
Supplement: Supplementary file 1 — Additional file 1. Definitions of reasons used to analyze unplanned emergency department return visits (URV) and categorization of the reasons. [file 12877_2023_4021_MOESM1_ESM.docx]

**Additional file 1.** Definitions of reasons used to analyze unplanned emergency department return visits (URV) and categorization of the reasons.

**Reasons for ED return per category Definition**

Patient-related ED return

Non-compliance There is evidence in the medical records that the patient did not follow instructions. The patient returned to the ED for the same problem.

Left without being seen The patient was registered in the ED but left before being seen by a physician. The patient returned to the ED for the same problem.

Left against medical advice The patient was seen by a physician and left the ED against medical advice. The patient returned to the ED for the same problem.

Patient was instructed to visit own GP The patient was instructed to return to the GP for re-evaluation but did not go and returned to the ED instead.

Psychiatric disorder / substance abuse The patient has a psychiatric disorder and/or uses drugs or alcohol, which causes him/her to repeatedly visit the ED for the same or similar problems. Mentally, the patient is in a chronic stable state.

Worrying about health The patient’s anxiety caused him/her to return to the ED for the same or similar problem. After re-evaluation in the ED, there was no change in diagnosis or treatment and medical management consisted of reassurance only.

Patient refuses admission or treatment The patient refused the treatment advised by the treating physician during the index ED visit or refused hospital admission. The patient returned to the ED for the same problem.

Illness-related ED return

Recurrent complaints/disease The patient was diagnosed and treated appropriately during the index ED visit, with resolution of symptoms, but later returned with a second exacerbation of the disease or with recurrence of the same or similar problem.

Complication The patient was diagnosed and treated appropriately during the index ED visit, but returned to the ED because of a complication of the disease or side effect of treatment (e.g., allergic drug reaction).

Progression of disease The medical records reveal that the patient was treated appropriately at the index ED visit and that admission was not indicated. Appropriate follow-up was arranged, but the patient’s disease or problem got worse, and he/she returned to the ED as instructed.

Failure of adequate treatment The patient was diagnosed and treated appropriately during the index ED visit, but the symptoms did not resolve, neither progressed (e.g., persistent pain due to fracture despite adequate use of pain medication). The patient returned to the ED because of persistent complaints.

New complaint The patient returned to the ED with a new complaint, which was different from the disease or complaint presented at the index ED visit and not determined as a complication or different presentation of the disease, presented during the index ED visit.

Other

*Physician-related ED return*

Treatment error The physician made the right diagnosis during the index ED visit, but made an error in treatment. The patient returned to the ED for the same or similar problem or progression of disease.

No painkillers prescribed The disease or injury warranted pain medication but no prescription or advice for the use of pain medication was given. The patient returned primarily because of continued pain.

Misdiagnosis Medical record review reveals a diagnosis or problem missed by the physician who saw the patient during the index ED visit. The patient returned to the ED for the same problem.

Discharged while admission indication Medical record review reveals a hospital admission indication, considering the severity of the patient’s complaints, but the physician judged that admission was not indicated. The patient returned to the ED because of the severity of the complaints.

*System-related ED return*

Not admitted due to lack of

hospital capacity Hospital admission was indicated, but the patient was sent home due to lack of hospital admission capacity. The patient returned to the ED for the same problem.

Not called for follow-up The patient did not receive a follow-up appointment within the time limit set upon discharge after the index ED visit, due to system-related reasons (e.g., miscommunication, waiting list). The patient returned to the ED for the same problem or progression of disease.

*Undefined ED return* The reason for the patient’s return cannot be classified in one of the other reasons for an URV.

*ED* Emergency Department, *e.g.*, exempli gratia, meaning “for example”, *GP* General practitioner, *URVs* Unplanned emergency department return visits
